# Cryptographic triboelectric random number generator with gentle breezes of an entropy source

**DOI:** 10.1038/s41598-024-51939-2

**Published:** 2024-01-16

**Authors:** Moon-Seok Kim, Il-Woong Tcho, Yang-Kyu Choi

**Affiliations:** 1 School of Electrical Engineering, Korea Advanced Institute of Science and Technology (KAIST), 291 Daehak-ro, Yuseong-gu, Daejeon, 34141 Republic of Korea; 2 The Department of Semiconductor System Engineering, Hanbat National University, 125 Dongseo-daero, Yuseong-gu, Daejeon, 31538 Republic of Korea

**Keywords:** Devices for energy harvesting, Electrical and electronic engineering

## Abstract

A wind-driven triboelectric nanogenerator (W-TENG) is a promising energy harvesting device due to its clean, ubiquitous and unexhausted properties. In addition, a W-TENG induces unpredictable chaotic outputs from wind flow that can serve as an entropy source for cryptography. This can be applied to a true random number generator (TRNG) for a secured system due to its inherent turbulent nature; thus, a W-TENG with a two-in-one structure can simultaneously generate both power and true random numbers. However, a previously reported W-TENG had one major drawback: a wind velocity of 10 m/s is required for stable energy harvesting by wind force. Thus, it is timely to demonstrate a W-TENG-based RNG whose operating condition is below 3 m/s, which is a gentle breeze similar to natural wind. In this study, we demonstrate a wind-driven cryptographic triboelectric random number generator (WCT-RNG) by using a W-TENG whose operating condition for wind speed is below 3 m/s by adopting a rear-fixed film structure instead of a conventional structure. The rear-fixed film refers to the fluttering film being freestanding on the front-side and fixed on the rear-side, where the front- and rear-sides are the wind inlet and outlet, respectively. The WCT-RNG enables the W-TENG to operate below a 3 m/s wind velocity. Because of this, the working time of the WCT-RNG is dramatically enhanced from only 8–42% at an average altitude above sea level. As the capability of operating at low wind speeds is significantly improved, a WCT-RNG becomes more useful and practical for generating both power and true random numbers in a single device. The device can thereby lead to the construction of a self-powered TRNG and secure communication for Internet of Things (IoT) devices in various environments, even under a gentle breeze. In this study, we explain the design of a WCT-RNG structure and also evaluate its randomness by using an NIST SP 800-22 B test suite with a reliability test.

## Introduction

Recently, the Internet of Things (IoT) has emerged as a new computing paradigm that can connect devices, objects, machines, and people through hyper-connectivity^1,2^. Forecasts predict that the number of online IoT devices will exceed 64 billion by 2025, with devices installed in various locations^3^. In IoT and smart system technology, each device commonly provides services to maintain an unexhausted power supply and communicate with other devices^4–6^. To ensure the functioning of IoT systems, it is crucial to install primitives that guarantee security functions for all devices, as IoT security attacks have increased by 77% in 2022 compared to the previous year, totaling 57 million, according to the 2022 Cyber Threat Report by SonicWall^7^. To prevent security threats, each system must have security functions: (i) confidentiality, (ii) integrity, (iii) availability, (iv) authentication, and (v) non-repudiation^8^. A hardware-based security device, known as a true random number generator (TRNG), is essential for supporting the aforementioned security functions as compared to a software-based security primitive, which uses a specific algorithm to generate pseudo-random numbers and is vulnerable to attacks or exploitation^9,10^. Therefore, developing TRNGs for various devices and situations is crucial for achieving secure IoT technology^11,12^.

Previously, we demonstrated a TRNG using a prototyped wind-driven triboelectric nanogenerator (W-TENG)^13^. The W-TENG-based TRNG not only provides energy harvesting but also security functions for communication systems such as IoT, smart grids for electricity networks, and in-flight applications. It produces true random numbers by converting chaotic wind flow to a random electrical signal. We demonstrated that the electrical signals directly induced from the W-TENG exhibits randomness without the need of post-processing, which is an extra process to manipulate or transform the generated random numbers to meet specific requirements.

However, an operation condition of the abovementioned W-TENG prototype is limited to high wind velocities of over 10 m/s. For practical outdoor use, extending the applicable conditions of the W-TENG to operate in a natural gentle breeze is crucial. In this work, we propose a wind-driven cryptographic triboelectric random number generator (WCT-RNG) that harvests wind energy and generates random numbers under a gentle breeze. The proposed WCT-RNG where the front- and rear-sides are the wind inlet and outlet, respectively adopts the rear-fixed film structure. The WCT-RNG generates electricity with alternating current (AC) at low wind velocities, which is utilized for a random signal source. Unlike a conventional W-TENG with a 4-corner fixed structure that is actuated by strong vortex flow, the WCT-RNG can flutter with laminar flow as well as vortex shedding due to the freestanding nature of the rear-fixed fluttering film even for input wind with a relatively low velocity^14^. This WCT-RNG can help advance secured and self-powered IoT and smart mobile systems through its improved capability to operate using natural wind.

For comparative studies, two types of a conventional W-TENG were used as a control group. Control group I adopted the 4-corner fixed fluttering film structure of conventional W-TENG^13^ which is named 4FW-TENG. Control group II employed a decoupled rear-fixed film W-TENG (RFW-TENG) structure. Decoupled RFW-TENGs are systems where the upper and lower TENG units independently generate energy through a separated electrical load. Conversely, a coupled RFW-TENG as an experimental group generates energy through a single common electrical load, which is used for a proposed WCT-RNG.

## Materials and methods

### Fabrication of WCT-RNG

For this study, we fabricated a WCT-RNG which is fixed at the rear but freestanding at the front; the input wind comes in via the freestanding side and the output wind exits through the fixed side^14^. The freestanding part of the film at the front-side enables the film to flutter with both a laminar and vortex flow; thus, the WCT-RNG can operate at a lower wind velocity compared to conventional W-TENG and FW-TENG. The upper and lower plates were manufactured by 3D printing (3DWOX1 from Sindoh) composed of curable resin. Their sizes are fixed at a length (*L*) of 72 mm, a width (*W*) of 34 mm, and a height (*H*_PLATE_) of 3 mm with consideration of optimal power density^14^.

Aluminum (Al) with a thickness of 0.3 mm was attached to the inner surface of the exoskeleton resin at the upper and lower plates. Then, perfluoroalkoxy (PFA) film with a thickness of 50 μm was attached onto the abovementioned Al plates. Figure S1 describes the structural specifications for the fabricated WCT-RNG.

The fluttering film is composed of quintuple layers, starting from the top layer of nylon, followed by a layer of Al, then a layer of polyimide (PI), followed by another layer of Al, and finally, a layer of nylon at the bottom. Among these layers, the core film at the center is a PI film with a thickness of 50 μm. The design aims to amplify triboelectric effects through physical contact and separation between the PI film and Al electrode^15^. Al with a thickness of 30 nm was deposited onto both sides of the PI film using an evaporator: first on the front side, then on the backside. Afterwards, a nylon coating process was conducted onto the exterior of the Al films. Nylon has excellent homogeneity, mechanical robustness, and thermal stability^16,17^. Thus, nylon serves as a protection layer which can enforce mechanical robustness to the abrasion of the inner Al layer during iterative contact-separation for both the upper and lower plates and the fluttering film. Thus, the proposed WCT-RNG possesses excellent long-term endurance characteristics. The coating process of the nylon is as follows. First, Nylon 6 (Sigma-Aldrich) is dissolved by a solvent (chloroform: 2,2,2-trifluoroethanol = 1:1, v/v). Next, this Nylon 6 solution is spin-coated onto both sides of the Al film at 3000 rpm for 30 s. Finally, the fabricated fluttering film is baked at 150 °C for 10 min in an oven.

The fluttering film is installed between the upper plate and lower plate. The manufactured WCT-RNG has four wedge-structure protrusions, with two wedges on the top plate and two on the bottom plate, as well as two alignment pins on the front-side, as shown in Fig. 2a,b. The fluttering film was punctured to create two holes, with each pin passing through a hole. These two pins act as a stopper to prevent the fluttering film from getting caught inwardly by aligning it inside the punctured holes. Additionally, the WCT-RNG has four supports on the rear-side, with two on the top plate and two on the bottom plate, as shown in Fig. 2a,b. At each supporter, there are two magnets under the upper resin supporter and two magnets over the lower resin supporter. These supporters firmly fix the fluttering film at the rear-side. Note that the total height (*H*_TOTAL_) of the WCT-RNG is 18 mm, which is the sum of the height of the upper supporter (*H*_UP_SUP_ = 7.5 mm), the two upper magnets (*H*_UP_MAG_ = 1.0 mm), the thickness of the fluttering film with the quintuple layers (*H*_FLUTTER_ = 1.0 mm), the two lower magnets (*H*_LO_MAG_ = 1.0 mm), and the lower supporter (*H*_LO_SUP_ = 7.5 mm). Figure S1 describes the heights for the fabricated WCT-RNG. For quick optimization of the *H*_TOTAL_, two resin supporters were replaced by stacked magnets, as shown in Fig. 4 and Fig. S3. Thus, the *H*_TOTAL_ is controlled by changing the number of the stacked magnets.

These installed protrusions play a key role in making the fluttering film perform flip-flop actuation at a low wind velocity by facilitating the easy separation from the upper and lower plate, and the alignment pins prevent the fluttering film from curling inward toward the gap at a high wind velocity. Due to these unique structures, the WCT-RNG can work in a gentle breeze to a strong windstorm. In conclusion, the WCT-RNG can operate at a wind velocity of 3 m/s, at which no vortex shedding arises. Relevant dimensions of the WCT-RNG were optimized with reference to a wind velocity of 4 m/s. Moreover, the WCT-RNG can operate stably for more than 96 h at a high wind velocity of 30 m/s, which is comparable to the nominal wind velocity of a hurricane that can cause a human to be taken off their feet or make roof materials begin to come off.

### Applied wind pressure

To evaluate output performances from the WCT-RNG under laboratory environments, a wind speed regulator (SUS316L EP regulator) accompanied with speed measurement equipment (VT 115 and TESTO 512) was used to control a wind pressure in a range of 6 psi to 70 psi, equivalent to 3.0–30.0 m/s, respectively.

### Electrical measurements

The WCT-RNG was operated inside an aluminum shield box to screen out any external noisy electromagnetic field, which can influence on the output performances, such as randomness. The electrical outputs from the WCT-RNG were characterized using an electrometer Keithley 6514, which can directly measure electrical voltage and current with various ranges.

To evaluate long-term durability, a harsh wind velocity of 30 m/s was intentionally used for an acceleration test. For an acceleration test, we can intentionally impose harsh conditions. Nevertheless, the power harvested from stormy winds can be maintained even during natural and gentle breezes. It is important to note that the power generated from wind is proportional to the cube of the wind speed (*v*^3^)^18^. Therefore, the power output from the WCT-RNG under the condition of 30 m/s for 96 h can be comparable to that generated at 3 m/s for 10 years.

### Ethical approval

This article does not contain any studies with human participants or animals performed by any of the authors.

## Results and discussion

The proposed WCT-RNG with a two-in-one configuration simultaneously acts as a power generator and TRNG, providing significant merits as an RNG and energy harvester. The capability of operating at low wind speeds has been dramatically improved over conventional methods. Among the aforementioned five security functions, ‘availability’ refers to the time-based probability that a target device is working properly at any given time, i.e., the working-time probability quantified with percentage. In Fig. S2, a detailed method is described to extract availability from statistical wind data. Availability can be a concern when an entropy source arises not from artificial means such as manufacturing-induced variability and operational-induced fluctuation, but from nature, such as wind. Even more concerning is when the wind is too weak or blows intermittently. The improved availability of the WCT-RNG in this work is attributed to the lowered working wind velocity: from 10 m/s, corresponding to a moderate gale, to 3 m/s, which is equivalent to natural wind. Wind energy varies according to various environments, and there are many statistical analyses in terms of wind speed and energy^19–22^. In conclusion, the average wind velocity can be statistically extracted according to the altitude above sea level in terms of spatial distribution. Conversely, in terms of time evolution, wind speed forms Weibull statistical distribution^23–25^; thus, the availability for the proposed WCT-RNG, whose operating condition is below 3 m/s, can be estimated. The 4FW-TENG (control group I), which works at a wind velocity of 10 m/s, was used as a primary reference^13^.

**Figure 1:**
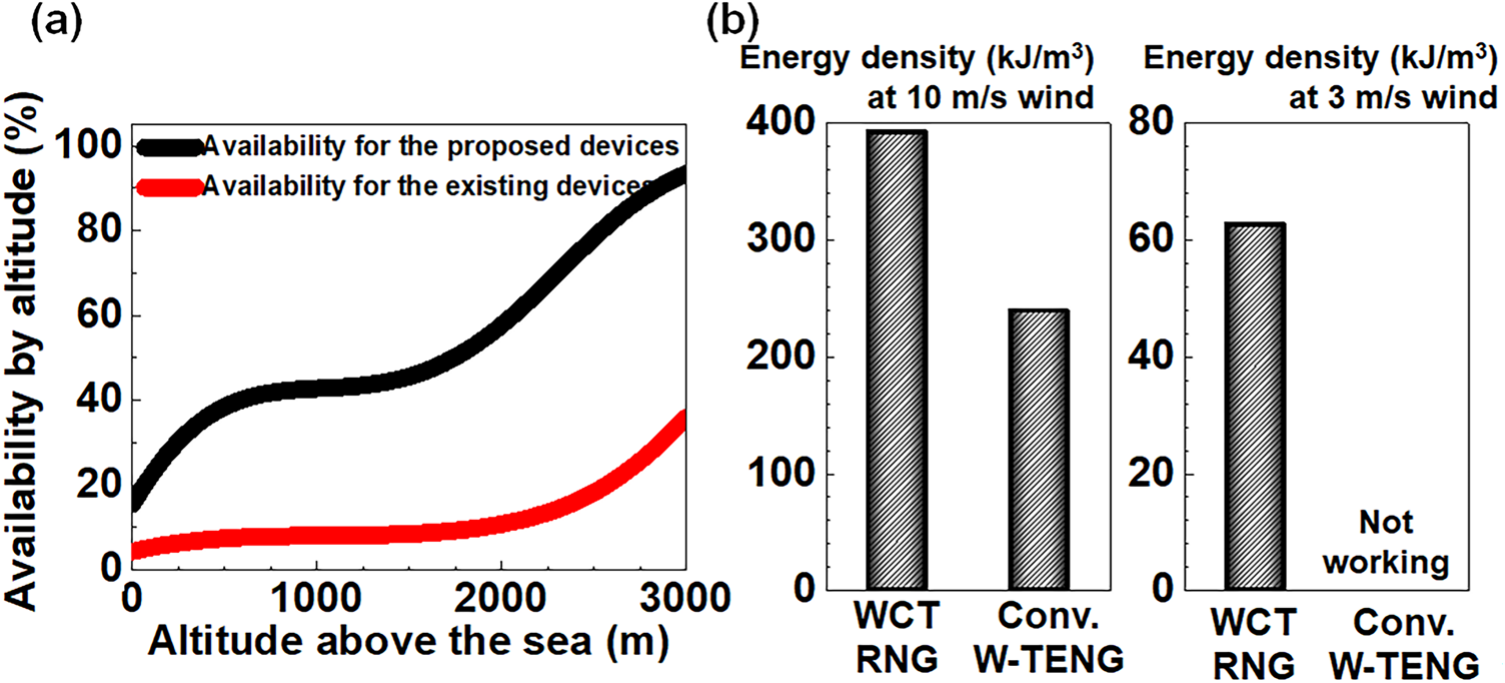
Comparison of the proposed WCT-RNG (experimental group) and conventional 4FW-TENG (control group II). (**a**) Compared availability between the proposed WCT-RNG and the conventional 4FW-TENG according to altitude above a sea level. (**b**) Compared histograms of estimated energy density at a wind velocity of 10 m/s and 3 m/s.

Figure 1a compares the availability for the proposed WCT-RNG (experimental group) and the conventional 4FW-TENG (control group I) according to the altitude above sea level. For example, the working-time availability for the proposed WCT-RNG is 42% at 840 m, which is the world’s average elevation^26,27^. This implies that the proposed WCT-RNG can generate energy and random numbers for 10 out of 24 h in a day. In contrast, the availability for the 4FW-TENG is 8% at 840 m, working only 2 out of 24 h. Thus, the working time for the proposed WCT-RNG is 5.2 times longer than that for the conventional 4FW-TENG. Figure 1b compares the extracted energy density for wind velocities of 10 m/s and 3 m/s. Specifically, it is defined as the harvested energy (*E*_harvested_) divided by the total volume (*L*⋅*W*⋅*H*_PLATE_). *E*_harvested_ was estimated by *V*_max_⋅*I*_max_⋅*T*_opr_, where *V*_max_ is maximal voltage, *I*_max_ is maximal current, and *T*_opr_ is operation time in a day. Here, both *V*_max_ and *I*_max_ were measured at load resistor (*R*_load_) of 60 MΩ. Maximum power was extracted at *R*_load_ of 60 MΩ from a WCT-RNG device^14^. At 10 m/s, *E*_harvested_ from the proposed WCT-RNG is 392.2 kJ/m^3^ in a day, which is 1.6 times larger compared with *E*_harvested_ from the 4FW-TENG. Even at 3 m/s, the WCT-RNG still produces 62.6 kJ/m^3^ in a day; however, the conventional 4FW-TENG does not work at all. Therefore, the proposed WCT-RNG is superior to the conventional 4FW-TENG in terms of working-time availability and power generation.

**Figure 2:**
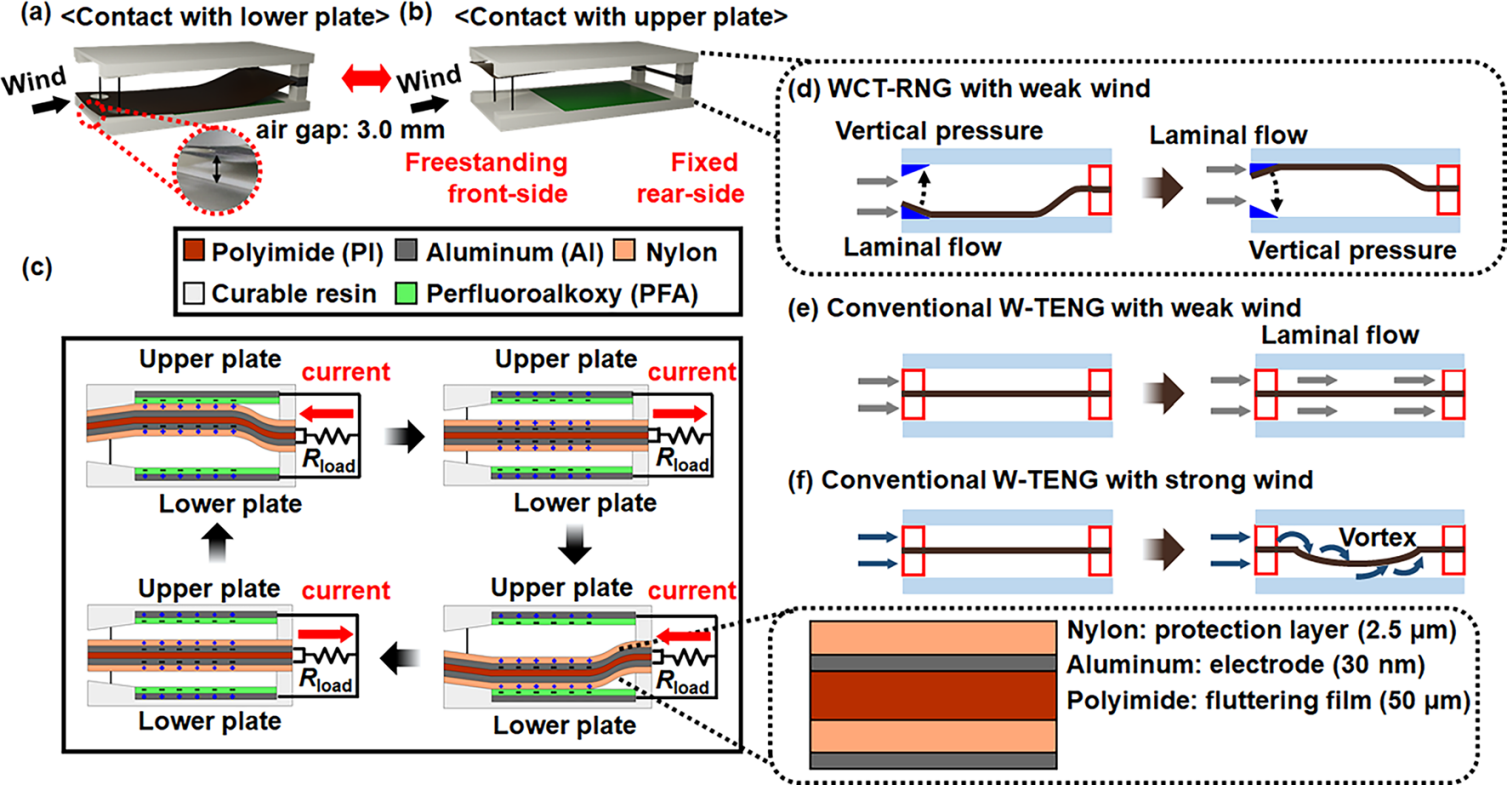
Schematic illustration of the proposed rear-fixed WCT-RNG for tilted and cross-sectional view. (**a**) Tilted view of the WCT-RNG where the fluttering film contacts the lower plate. (**b**) Tilted view of the WCT-RNG where the fluttering film contacts the upper plate. (**c**) Cross-sectional view of the WCT-RNG to show current flowing with a coupled mode that the WCT-RNG shares a common *R*_load_. (**d**) Cross-sectional view of the WCT-RNG working with weak wind. (**e**) Cross-sectional view of the conventional W-TENG not working with weak wind. (**f**) Cross-sectional view of the conventional W-TENG working with strong wind.

Figure 2a,b are schematic illustrations of the WCT-RNG when the fluttering film is in contact with the lower plate and upper plate, respectively. As a shim, wedge-shaped protrusions were implemented to separate the fluttering film slightly from the resin plate so it can easily move up and down, even in a gentle breeze. Moreover, the wedge is also a favorable structure that redirects the lateral wind pressure to vertical force acting on the fluttering film; thus, the fluttering film can vertically move up after contact between the film and lower plate, as shown in Fig. 2a. With the same principle, the fluttering film vertically moves down after contact between the film and upper plate, as shown in Fig. 2b. Without these wedges, the fluttering film cannot move up and down because the film adheres to the upper or lower plate, which is illustrated in Fig. S4a.

When the bendable film flutters up and down, the surface of the nylon on the fluttering film is positively charged and the surface of the PFA on the resin plate is negatively charged via contact electrification^28,29^. The upper TENG unit creates electrical power when the fluttering film contacts and separates from the upper plate via electrostatic induction. Specifically, electrical current flows from the electrode of the upper plate to the upper electrode of the fluttering film when the film comes into contact with the upper plate. Conversely, when the film separates from the upper plate, electrical current flows from the upper electrode of the fluttering film to the electrode of the upper plate. The lower TENG unit also generates electrical power through the same principle that operates in the upper TENG unit.

Figure 2c exhibits the cross-sectional configurations of the WCT-RNG while describing the transient electrical voltage and current behaviors according to the movement of the fluttering film, like one period of a sine wave, as shown in Fig. S3a. This is because the fluttering film has two fixed ends. In contrast, the fluttering film of the 4FW-TENG moves like a quarter period of a sine wave owing to the end structure of the fluttering film, i.e., the curved shape of the fluttering film by wind is just concave or convex, as shown in Fig. S3b. Thus, the 4FW-TENG has a single unit unlike the double unit in the WCT-RNG.

As long as a chaotic wind is introduced to a gap of the WCT-RNG, the fluttering film contacts the upper plate then the lower plate iteratively or vice versa. When the fluttering film contacts the upper plate, current flows from the upper plate to the fluttering film through *R*_load_. Afterwards, as they are far apart from each other, current flows reversely from the fluttering film to the upper plate through the *R*_load_. Thereafter, the fluttering film contacts the lower plate, and in turn, current flows from the lower plate to the fluttering film through the *R*_load_. Then, when they are separated, current reversely flows from the fluttering film to the lower plate through the *R*_load_. Thus, the WCT-RNG inherently has a pair of electrodes: an upper TENG and a lower TENG that are connected in parallel. Each fluttering motion from each TENG cannot be identical due to turbulent wind in the gap that is continuously changing from time to time, even though the two TENGs are the same as each other. Consequently, the simple arithmetic sum of each open-circuit voltage (*V*_OC_) from each decoupled TENG (control group II) cannot be identical to the total *V*_OC_ from the coupled TENGs, as compared in Fig. 5d,e. They may appear similar at first glance, however, they are different upon closer inspection. Herein, *V*_OC_ refers to the electrical potential value where the resistance value of the load is infinite. This chaotic coupling can make an auto-correlation coefficient (*R*_XX_ (*t*_1_,*t*_2_)) between times *t*_1_ and *t*_2_ rapidly decrease to 0; thus, it can improve the randomness of a set of generated random numbers, as explained later.

Figure 2d describes the movement of the film in the WCT-RNG, even for a gentle breeze. The protruded wedges redirect wind flow, thereby making a laminar flow induce vertical pressure on the freestanding fluttering film. However, the relative dimension of the stoppers with a diameter of 0.5 mm is negligibly narrow compared to the plate width of 3 cm; thus, it cannot significantly influence the airflow near the wind inlet. Without the stoppers, the freestanding fluttering film can roll in due to strong wind input, as shown in Fig. S4b.

Figure 2e,f describe the movements of films in the conventional W-TENG (4FW-TENG) with weak wind and strong wind, respectively. Unlike the WCT-RNG working at a wind velocity of 3 m/s, the fluttering film of the 4FW-TENG was flipped up and flopped down when a strong wind velocity of 10 m/s was applied. This is because weak wind flow cannot produce vertical pressure to drive the film movement in the 4FW-TENG owing to the front-side fixed and rear-side freestanding structure^14^. Conversely, the proposed WCT-RNG produces vertical pressure from weak wind flow to activate film movement by the aid of the rear-fixed structure.

**Figure 3:**
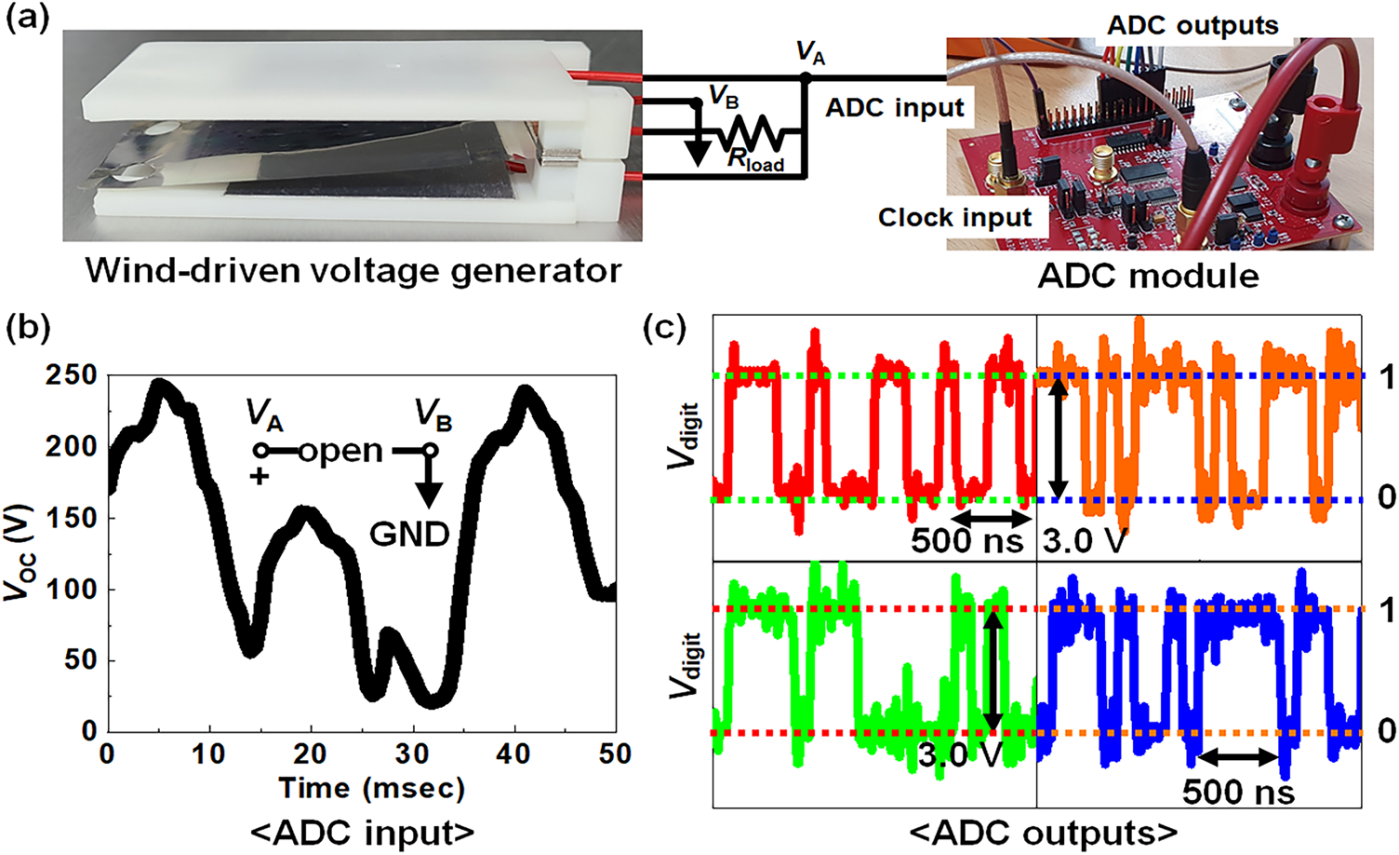
Configuration of 100% hard-ware based TRNG and its electrical outputs. (**a**) Optical photograph of manufactured WCT-RNG connected with an analog-to-digital converter (ADC) hardware (ADC-08100) module. (**b**) Measured analog output voltage (open-circuit voltage, *V*_OC_) from the WCT-RNG at a wind pressure of 8 psi (4 m/s). (**c**) Measured digital output voltage (*V*_digit_) from 4 pins of the ADC module for each digital pin.

Figure 3a shows an optical photograph of the fabricated two-in-one WCT-RNG enclosing a TENG as well as an RNG and the assembled analog-to-digital converter (ADC) module. The generated AC-typed voltage from the fabricated WCT-RNG shown in Fig. 3b is converted into digital signals by the ADC module, as shown in Fig. 3c. An ADC-08100 evaluation module (EVM) was used to convert the analog-typed *V*_OC_ to digital random bits^30^. These converted digital bits are used as true random numbers. First, wind energy is converted to analog electrical voltage in the form of *V*_OC_ through the WCT-RNG. Second, the analog output *V*_OC_ is quantized and sampled to produce 8-bit digital signals. These digital signals are temporarily stored in memory devices in the processing unit. Finally, random data can be generated from this stored data whenever an end user requests random numbers^31,32^. In conclusion, the WCT-RNG module provides random numbers from wind energy, which can be used in cryptographic protocols that provide functions such as confidentiality, integrity, and authentication^33,34^. More specifically, a set of the generated true random numbers can be used as a cryptographic key and a cryptographic nonce during encrypting and decrypting operations to guarantee security confidentiality^35,36^.

**Figure 4:**
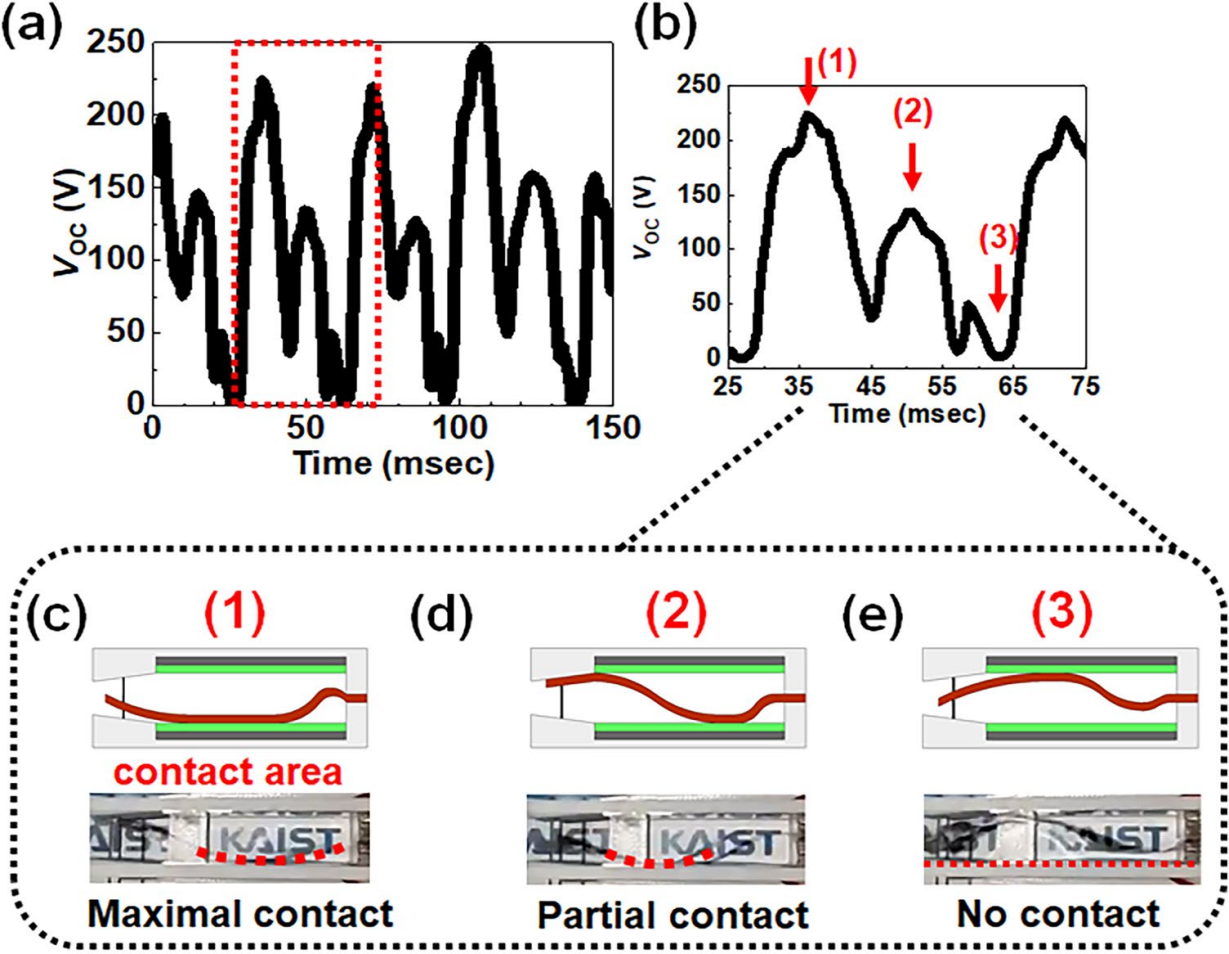
Measured *V*_OC_ with 4 m/s wind velocity at different contact positions. (**a**) *V*_OC_ extracted from the WCT-RNG. (**b**) Close-up view of the *V*_OC_ for the red box in (**a**). (**c**) Cross-sectional schematic and its optical photograph showing maximal contact, (**d**) showing no contact, and (**e**) showing partial contact.

The directly measured *V*_OC_ from the fabricated WCT-RNG is shown in Fig. 4a. Figure S5a exhibits the schematic illustration for characterization of *V*_OC_ and measured *V*_OC_, while Fig. S5b shows the schematic illustration for characterization of short-circuit current (*I*_SC_) and measured transient *I*_SC_. Its amplitude was 250 V at an input wind velocity (*v*_in_) of 4 m/s, and its close-up view is shown in Fig. 4b. The transferred charge (*Q*_TR_) was approximately 30 nC, which is extracted by integration of the measured *I*_SC_ with respect to time. Figure 4c–e display optical photographs and their corresponding schematics of the rear-fixed fluttering film in the manufactured WCT-RNG according to each peak position of *V*_OC_ in Fig. 4b. In one cycle period, there are three *V*_OC_ peaks. The highest *V*_OC_ peak is generated when the fluttering film is fully contacted to an electrode, as shown in Fig. 4c. The intermediate *V*_OC_ peak is created when the fluttering film is partially touching an electrode, as shown in Fig. 4d. The lowest *V*_OC_ peak is produced when the fluttering film is not in contact with the electrode, as shown in Fig. 4e. The *V*_OC_ with 3 peaks from the WCT-RNG looks like much more irregular, as *V*_OC_ with a single peak from the 4FW-TENG is like a half cycle of a sine wave^13,37^. In conclusion, the WCT-RNG generates a *V*_OC_ that exhibits more irregular amplitude with atypical periodicity compared with the 4FW-TENG.

**Figure 5:**
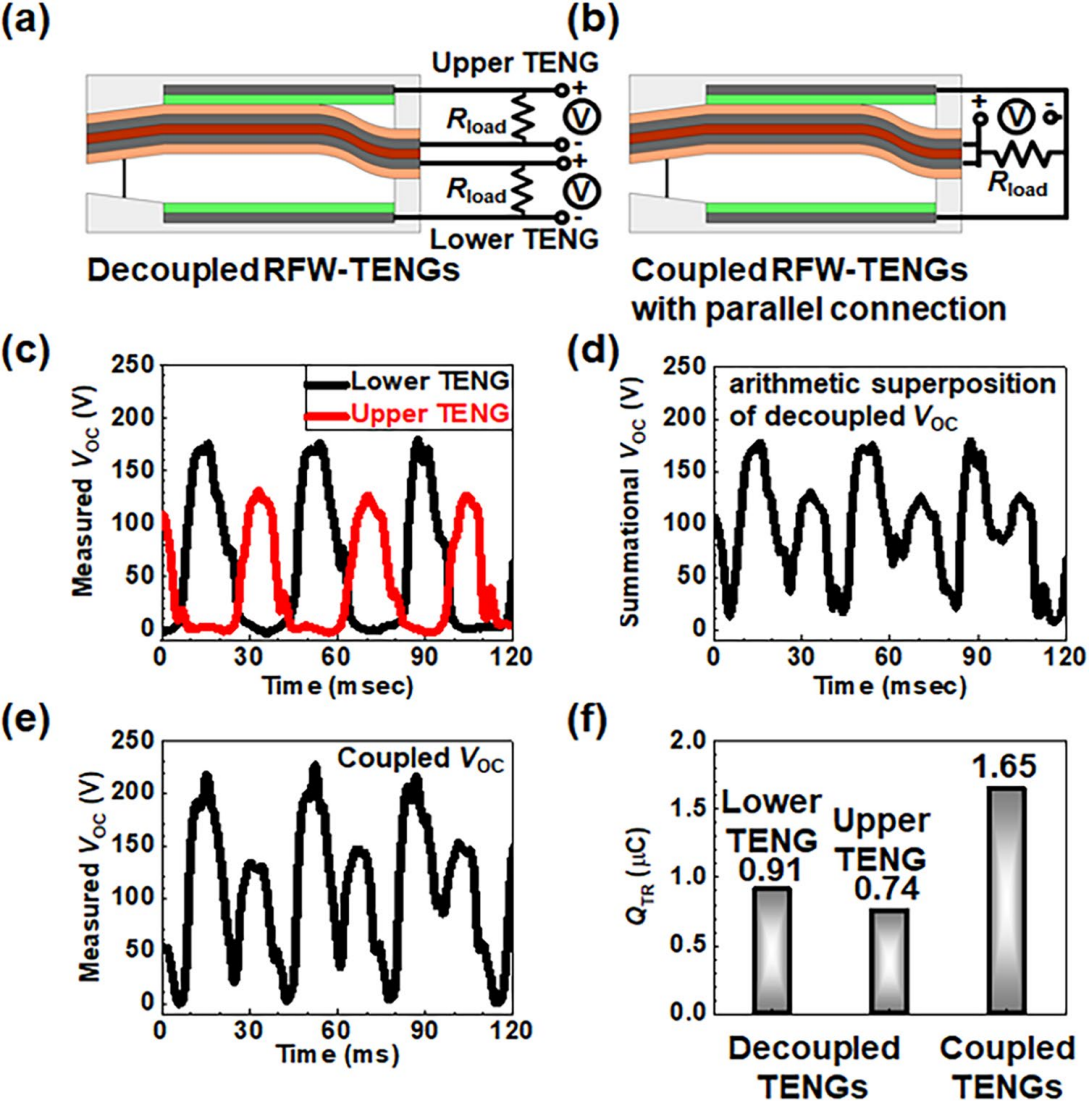
Cross-sectional schematics of the decoupled and coupled RFW-TENG and their characteristics. (**a**) Decoupled RFW-TENG (control group II) composed of the upper and lower TENG that have their own separated *R*_load_. (**b**) Coupled RFW-TENG (experimental group) comprised of the upper and lower TENG that shares a common *R*_load_. (**c**) Superimposed *V*_OC_ from the lower unit (black) and the upper unit (red) in the decoupled mode. (**d**) Arithmetically summed *V*_OC_ from the lower unit (black) and the upper TENG (red) in the decoupled mode. (**e**) Measured *V*_OC_ directly from the coupled RFW-TENG. (**f**) Comparison of *Q*_TR_ from the upper and lower TENG of the decoupled RFW-TENG and the coupled RFW-TENG.

Figure 5 compares the electrical characteristics between the decoupled and coupled RFW-TENGs. While the decoupled RFW-TENG is a control group II, the coupled RFW-TENG is an experimental group, i.e., the WCT-RNG. In the decoupled RFW-TENG, the upper and lower TENG unit independently generates energy through the *R*_load_. Thus, the experimental data of control group II shows two different periodic signals of *V*_OC_, which can be superimposed later.

Figure 5a shows a schematic illustration of the decoupled TENG between the upper and lower TENG, which has its own *R*_load_. Each TENG unit independently generates each *V*_OC_ through the separated *R*_load_. In contrast, Fig. 5b depicts a schematic of the coupled TENG between the upper and lower TENG, which share a single *R*_load_. As the fluttering film moves up and down like a sine wave, both TENGs produce jointed *V*_OC_ via the common *R*_load_. Figure 5c exhibits a graph superimposing one *V*_OC_ from the upper TENG and the other* V*_OC_ from the lower TENG. However, Fig. 5d displays the arithmetically summed *V*_OC_ from the graph of Fig. 5c. The parallel connection of each decoupled TENG can make an arithmetic superposition of both outputs from the upper and lower TENG. A peak of the *V*_OC_ from the lower TENG is higher than that from the upper TENG due to downward gravitational force. Furthermore, Fig. S6 compares the measured *V*_OC_ for the upright RFW-TENG and the reversed RFW-TENG when used as an upside-down structure which verifies that the difference in the *V*_OC_ peak between them is attributed to gravitational force.

Conversely, Fig. 5e shows the directly measured *V*_OC_ from the coupled RFW-TENG through the shared *R*_load_ from the graph of Fig. 5c. In the case of the decoupled mode, the arithmetic sum between each TENG is evaluated after the calibration with an intentionally coherent phase, allowing for a direct comparison with the measured signals. Even though the overall waveform of Fig. 5d is similar to that of Fig. 5e, they are not the same upon careful examination. The similarity between Fig. 5d and e indicates that the total *V*_OC_ of the RFW-TENG is composed of each *V*_OC_ from the upper and lower TENG, and there are three notable features. First, the *V*_OC_ for both the upper and lower TENG possess periodic characteristics with approximated time intervals of 40 ms. Second, the *V*_OC_ measured from both the upper and lower TENG has complementary characteristics, meaning the time intervals to show peak voltage do not overlap. In other words, the high-voltage regions between the black line and red line in Fig. 5c do not overlap due to the fluttering film hitting the upper and lower TENG in rotation. Third, each amplitude of the *V*_OC_ from the upper TENG and the lower TENG is slightly different due to the intrinsic structure, where the contact force is stronger when the fluttering film goes to the lower unit than the upper unit by downward gravitational force. Figure 5f shows the amount of *Q*_TR_ per second from wind energy. The summation of each *Q*_TR_ from each decoupled TENG is the same as the measured *Q*_TR_ from the coupled TENGs, even though the amplitude and period of the *V*_OC_ are not identical. In conclusion, the signal of proposed WCT-RNG devices is composed of two *V*_OC_. One is from the upper TENG and the other is from the lower TENG. It should be noted that the coupled *V*_OC_ through a common electrical load is more random compared with each *V*_OC_ via a separated electrical load.

**Figure 6:**
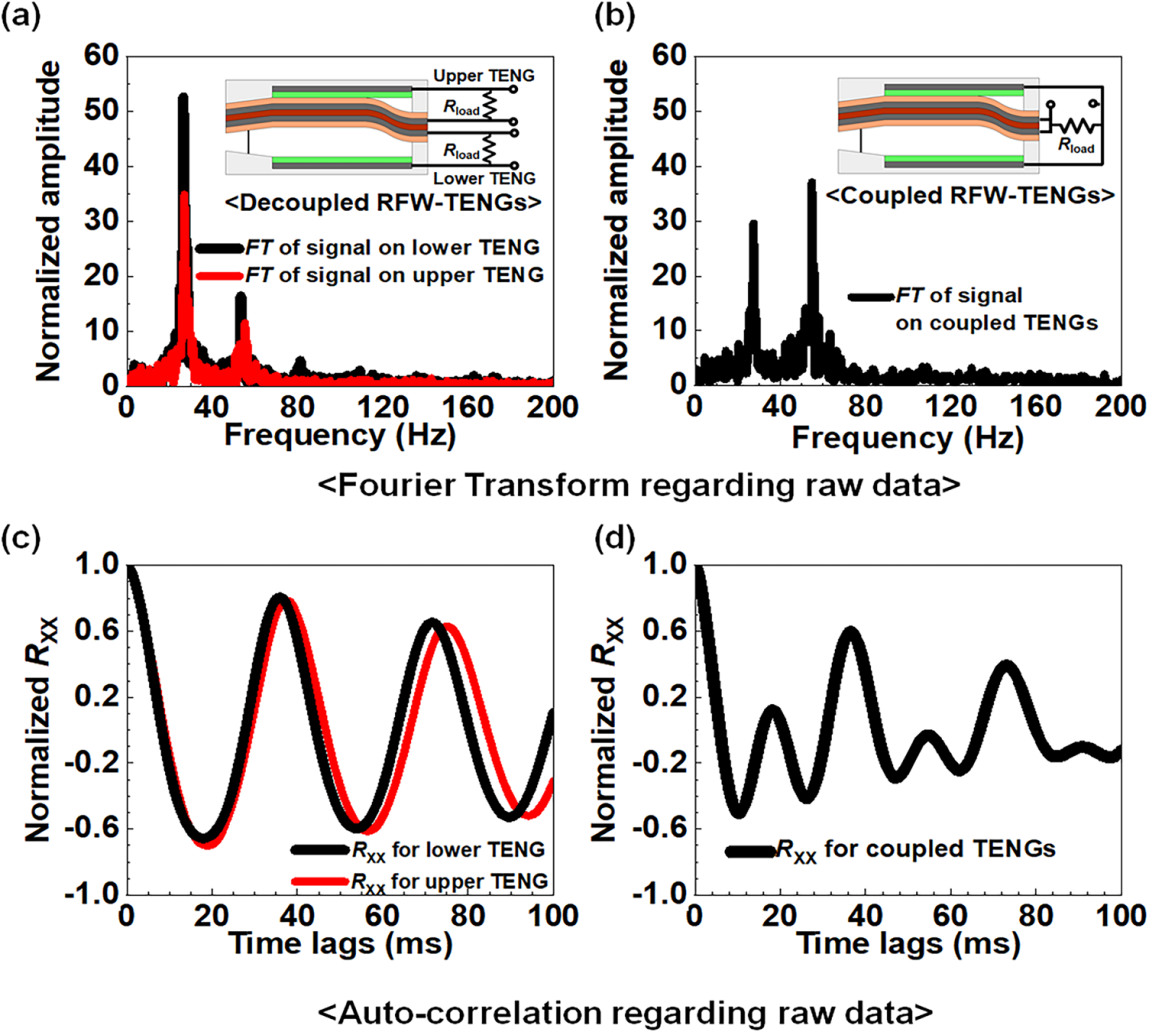
Comparison of discrete Fourier transform (FT) data and the auto-correlation coefficient (*R*_XX_) between the decoupled mode and a coupled mode. (**a**) Superimposed FT spectrum of the measured *V*_OC_ from the lower (black line) and upper TENG (red line) of the decoupled RFW-TENG (control group II). (**b**) FT spectrum of the measured *V*_OC_ from the coupled RFW-TENG (experimental group). (**c**) Superimposed *R*_XX_ of the measured *V*_OC_ from the lower (black line) and upper TENG (red line) of the decoupled RFW-TENG. (**d**) *R*_XX_ of the measured *V*_OC_ from the coupled RFW-TENG.

Figure 6a,b show the normalized amplitude after the discrete Fourier transform (FT) of the *V*_OC_ from a time domain to frequency domain for the decoupled and coupled RFW-TENGs, respectively^38,39^. Figure 6a shows the output results of the FT according to the frequency on the lower TENG (red line) and upper TENG (black line) in the case of the decoupled RFW-TENG. Conversely, Fig. 6b denotes the output results of the FT in the case of the coupled RFW-TENG. All signals in Fig. 6a,b possess identical spectrum peaks: 27.5 Hz and 55.0 Hz. Conventional W-TENGs that operate only at high velocity wind exhibit electrical signals with characteristics of a single-frequency system^13,37^. Thus, the signals that conventional W-TENGs produce are modeled as sinusoidal waves that possess Gaussian noise distribution for amplitude and frequency^13^. A single-frequency system is represented as *S* = *A*_0_‧cos(2π‧*f*_0_‧*t*), where *A*_0_ is the amplitude of the cosine signal, *f*_0_ is the frequency for a single frequency system, and *t* is the transient time. However, the proposed RFW-TENG, which operates under low-velocity wind conditions, possesses a dual frequency system with dual peak frequencies of 27.5 (= 55.0/2) Hz and 55.0 Hz. These frequencies indicate that the measured transient signals are approximately represented to *S* = *A*_1_‧cos(2π‧55*t*/2) + *A*_2_‧cos(2π‧55*t*), where *A*_1_ is the amplitude of the cosine signal with 27.5 Hz, *A*_2_ is the amplitude of the cosine wave with 55.0 Hz, and *t* is the transient time. Figure S7 shows the supposition of the sinusoidal signal between the *f*_1_ = 27.5 Hz and *f*_2_ = 55.0 Hz. It can be inferred that the signals produced by the dual peak frequency system are more complex than those produced by the single-frequency system. The dual peak frequencies originate from the dual contacts between the fluttering film and each electrode during one cycle. Figure S3a shows a snap-shot photograph of the fluttering film contacting the upper plate twice per cycle. Figure S3b displays a snap-shot photograph of the film of conventional W-TENG contacting the upper plate once per cycle. Due to these multiple contacts, the proposed WCT-RNG possesses a smooth fluttering movement in the fluttering film, unlike than that of conventional W-TENG, which is supported by Fig. S3a,b.

Figure 6c,d represent the auto-correlation coefficient (*R*_XX_) of the decoupled RFW-TENG and the coupled RFW-TENG, respectively. The *R*_XX_ refers to the self-similarity of the signal over different delay times, i.e., the correlation of a signal with a delayed copy of itself as a function of delay^40^. Because it is useful to know how many repeating patterns there are, the *R*_XX_ can be a well-known parameter to visually estimate randomness^41^. The *R*_XX_ ranges from − 1 to 1. A high correlation of the |*R*_XX_| close to 1 indicates a uniform signal, whereas a low correlation of the |*R*_XX_| close to 0 presents a non-uniform and atypical signal, i.e., no correlation between two values of the same variable but at different times^42^. The *R*_XX_ shown in Fig. 6d reduces more rapidly than that in Fig. 6c according to time lag. The rapid reduction indicates that there is no relationship with a self-delayed signal^43,44^. Thus, the coupled RFW-TENG produces random numbers with improved randomness compared to the decoupled RFW-TENG. Therefore, from a TRNG point of view, the WCT-RNG is superior to both the decoupled RFW-TENG (control group II) and the previously reported 4FW-TENG (control group I)^13,37^.

Table 1 compares the pass rate for each sub-suite test of the NIST SP 800-22 B for the evaluation of randomness^45,46^. The pass rate refers to the probability the test sequence satisfying the condition of a *p*-value ≥ *α* (significance value), while the significance level of *α* was set to 0.01. A recommended significance value by NIST ranges from 0.001 to 0.01^46^. To evaluate randomness for all 15 sub-suite tests, a bit stream of 4,000,000 bits was directly extracted from the ADC module connected with the WCT-RNG. It shows an excellent pass rate of over 98% for all sub-suite tests. Conversely, the decoupled RFW-TENG (control experiment) exhibits a relatively low randomness. The average pass rate for the lower and upper TENG was 75.0 and 72.0, respectively. The complex electrical signals of WCT-RNG, originating from dual frequency characteristics with various noise frequencies and low correlation characteristics according to time lags, improve pass rate. In conclusion, the signal of the proposed WCT-RNG generates high-quality random numbers even when a low-velocity wind flow is applied.

**Table 1:** Comparison of each pass rate for all 15 test suites of the NIST SP 800-22 B for the decoupled mode (control group II) and WCT-RNG (experiment group).

Pass rate (%)	4,000,000 bits (significance = 0.01)		
	Decoupled RFW-TENGs		WCT-RNG
	Lower TENGs	Upper TENGs	
Frequency	91	78	98
Frequency within a block	100	100	100
Runs	93	93	99
Longest run of ones	90	95	98
Binary matrix rank	99	99	100
Discrete Fourier transform	89	89	100
Serial	85	0	98
Approximate entropy	99	0	100
Cumulative sums	89	0	99
Non-overlapping template	0	100	100
Overlapping template	0	77	100
Maurer’s universal statistics	0	98	100
Linear complexity	100	76	100
Random excursions	90	75	100
Random excursions variation	100	100	100
Average	75.0	72.0	99.5

**Figure 7:**
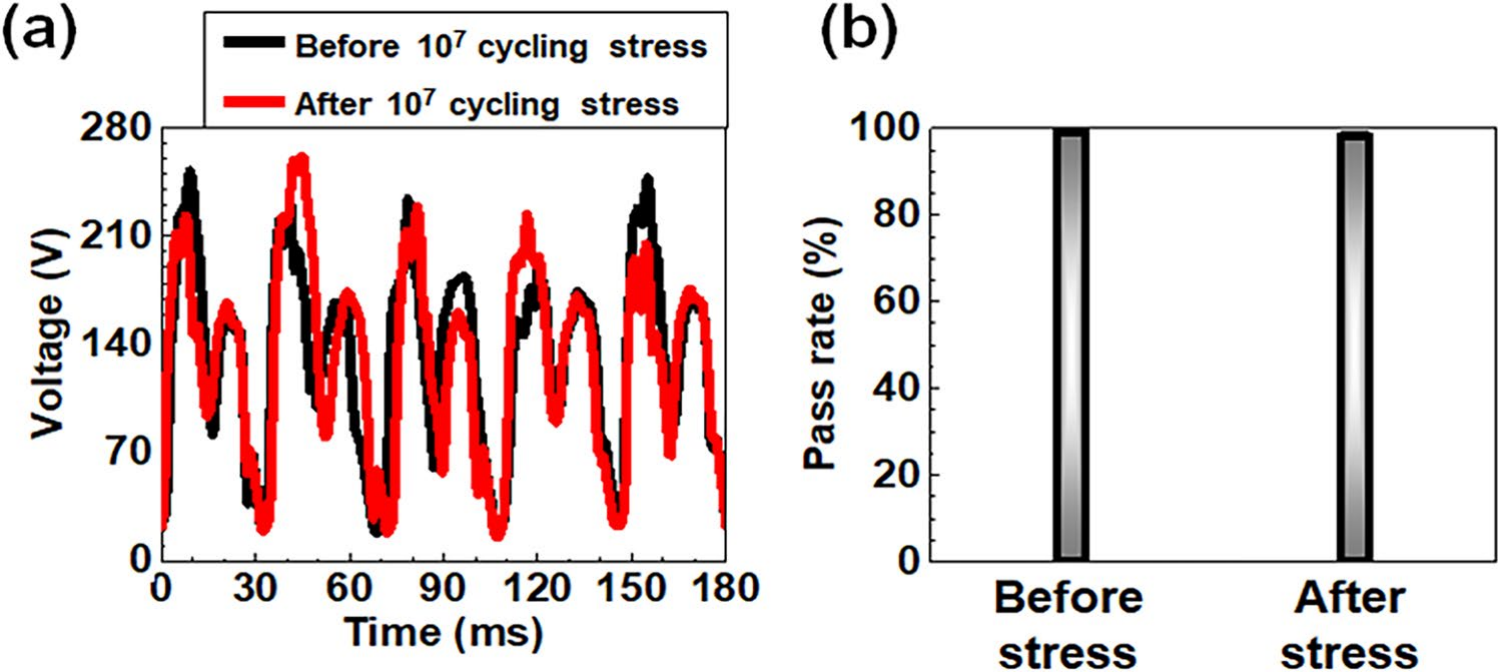
Comparison of TRNG performances in terms of durability. (**a**) Comparison of measured *V*_out_ from the RFW-TENG with a wind pressure of 8 psi (4 m/s) before and after cyclic stress of 10^7^ under an air pressure of 70 psi (30 m/s). (**b**) Comparison of average pass rate for the 9 brief test suites of the NIST SP 800-22 B between a fresh WCT-RNG and an aged WCT-RNG.

Figure 7a compares the transient electrical signals between a fresh WCT-RNG and an aged WCT-RNG after undergoing intentional iterative stresses. To confirm the durability of the manufactured WCT-RNG, repeated cyclic stresses of 10^7^ were intentionally applied with a wind pressure of 70 psi, which is equivalent to 30 m/s: comparable to the velocity of hurricanes and typhoons corresponding to Beaufort force level-12^47,48^. This harsh stress condition was used for an accelerated test to reduce the evaluation time of the durability within a short time. There was no degradation in the transient voltage even after cyclic stress. Figure 7b compares the averaged pass rate for the case of before stress and after stress. The pass rate was evaluated with a relatively shortened bit stream to further reduce the assessment time according to the 9 brief test suites of NIST SP 800-22 B^45,46^. The averaged pass rate was 99.5% for both cases and was unaffected even after the number of cyclic stresses up to 10^7^. We conclude that the proposed WCT-RNG can reliably work as an RNG against mechanical durability.

## Conclusion

We demonstrated a 100% hardware-based wind-driven cryptographic triboelectric random number generator (WCT-RNG) that utilizes a gentle breeze as an entropy source. This WCT-RNG consists of both an upper and a lower TENG, making it a two-in-one device as it serves as both an energy harvester and a true random number generator. Notably, the generated random numbers exhibited higher levels of randomness when the upper and lower TENG were in the coupling mode compared to the decoupling mode. In terms of randomness, the manufactured WCT-RNG exhibited a pass rate of 99.5% across all 15 test suites of the NIST SP 800-22B at 4 m/s. In terms of endurance, it maintained a 99.5% pass rate for the 9 brief tests of the NIST SP 800-22B, even after enduring 10^7^ iterative stresses at 30 m/s, which is equivalent to the stress of 3 m/s over a period of 10 years. Practicality can be further enhanced by integrating various components used in the current work into a single entity. This approach can pave the way for the development of self-powered and self-security functions in the era of IoT.

### Supplementary Information

Supplementary Information.

## Data Availability

The datasets generated during and/or analysed during the current study are available from the corresponding author on reasonable request.
