# Supplementary material for: Cryptographic triboelectric random number generator with gentle breezes of an entropy source
Source: Sci Rep. 2024 Jan 16;14:1358. doi: 10.1038/s41598-024-51939-2 (PMC10791744; doi:10.1038/s41598-024-51939-2)
Supplement: Supplementary file 1 — Supplementary Information. [file 41598_2024_51939_MOESM1_ESM.docx]

**Supplementary Information**

**Cryptographic triboelectric random number generator with gentle breezes of an entropy source**

Moon-Seok Kim^1,2§^, Il-Woong Tcho^1§^, and Yang-Kyu Choi^1^*

^1^ School of Electrical Engineering, Korea Advanced Institute of Science and Technology (KAIST), 291 Daehak-ro, Yuseong-gu, Daejeon 34141, Republic of Korea

*^2^* The Department of Semiconductor System Engineering, Hanbat National University, 125 Dongseo-daero, Yuseong-gu, Daejeon 31538, Republic of Korea

^§^M.-S. Kim and I.-W. Tcho equally contributed to this work.

* Correspondence and requests for materials should be addressed to Y.‒K. Choi ([ykchoi@ee.kaist.ac.kr](mailto:ykchoi@ee.kaist.ac.kr))

**1.** **Structure of proposed devices**

Figure S1a and S1b show the overall photographs of a manufactured WCT-RNG actuated by gentle breezes, because a fluttering film positioned in between an upper electrode (plate) and lower electrode (plate) is freestanding at the front-side and fixed at the rear-side. The white-colored upper and lower plates were manufactured with curable resin by 3D printing. The height of each plate (*H*_PLATE_) is 3mm, while the length (*L*) and width (*W*) of each plate are 72 mm and 34 mm, respectively. The fluttering film was strongly affixed at both corners of the rear-side. At each corner, the fluttering film was firmly fixed by magnetic attraction between two upper magnets at the top and two lower magnets at the bottom, as shown in Figure S1. The total height (*H*_TOTAL_) of the WCT-RNG is 18mm, which is the sum of the height of the upper supporter (*H*_UP_SUP_ = 7.5 mm), two upper magnets (*H*_UP_MAG_ = 1.0 mm), the thickness of the fluttering film composed of five layers (*H*_FLUTTER_ = 1.0 mm), two lower magnets (*H*_LO_MAG_ = 1.0 mm), and the lower supporter (*H*_LO_SUP_ = 7.5 mm).


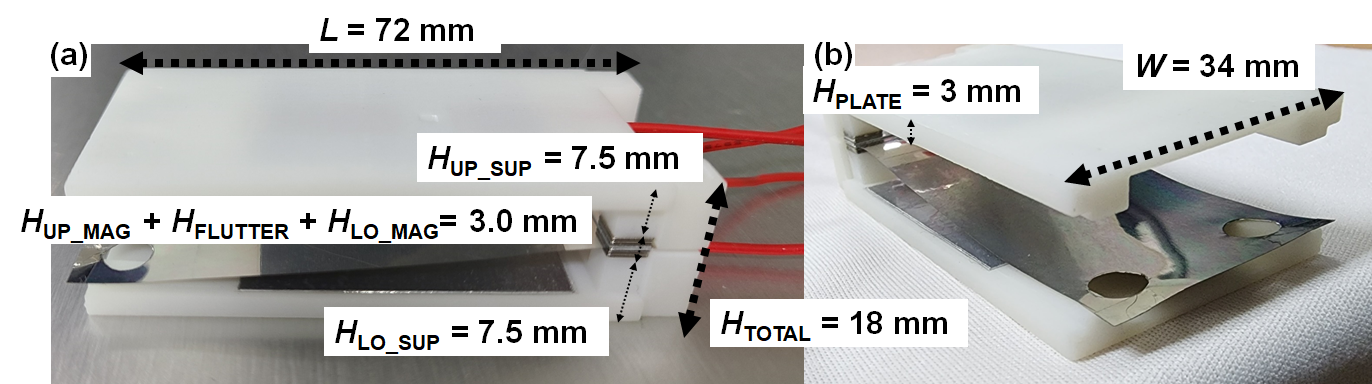


**Figure S1.** Optical photographs of the manufactured WCT-RNG. (a) Side view of the WCT-RNG. (b) Tilted bird’s-eye view of the WCT-RNG.

**2. Procedure to extract availability of RFW-TENG for various altitudes**

Figure S2 displays the procedure to extract availability according to an altitude (*z*), which is referenced to the sea level. Availability refers to the time-based probability that a target device is working properly at any given time, *i*.*e*., the working-time probability quantified by percentage. Based on previous statistical studies, availability was extracted for the WCT-RNG (experimental group) with a fluttering film fixed at only 2 corners and the 4FW-TENG (control group I) with a fluttering film fixed at all 4 corners. First, the average wind speed according to the altitude was extracted [1, 2]. Next, the probability density function according to wind speed was also extracted from statistical data such as averaged wind speed (*v*_average_). It is well known that the distribution of wind speed follows Weibull distribution [3, 4, 5]. This indicates that the probability density function according to wind speed can be represented by the Weibull distribution at the same position. From the distribution, the availability of the WCT-RNG and the 4FW-TENG is extracted. As a consequence, the availability of the WCT-RNG is approximately 4-fold larger than that of the 4FW-TENG.


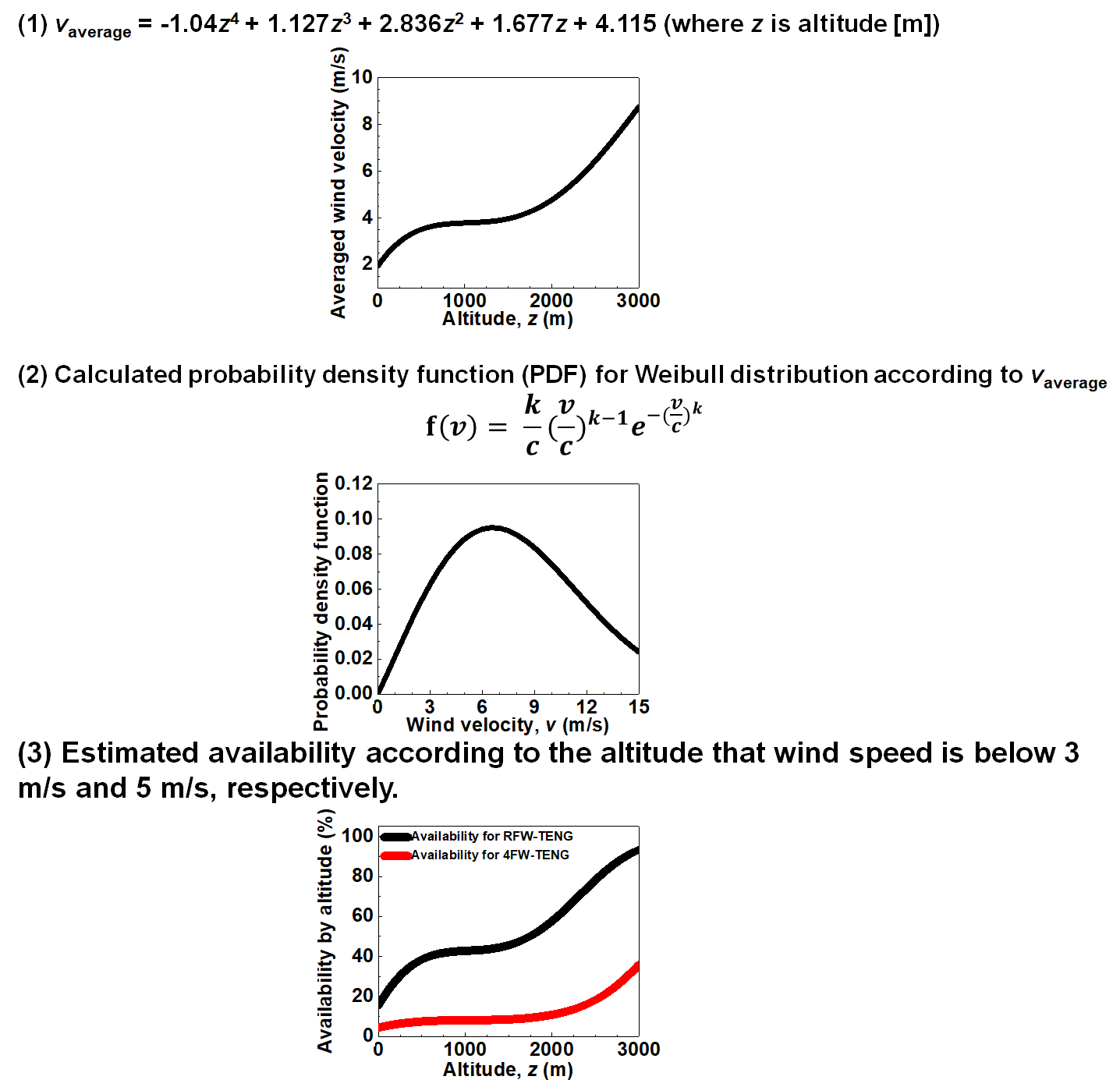


**Figure S2.** Procedure to extract availability for WCT-RNG (experimental group) and 4FW-TENG (control group I).

**3. Comparison of snapshot photographs between RFW-TENG and 4FW-TENG**

Figure S3a shows snapshot photographs of the manufactured WCT-RNG (experimental group), while Fig. S3b exhibits snapshot photographs of the 4FW-TENG (control group I) from recorded videos. Figure S3a displays the fluttering film of the WCT-RNG moving like a full-cycle sinewave, whereas S3b presents the fluttering film of the 4FW-TENG moving like a half-cycle sinewave.

In the case of the WCT-RNG, there are three peaks and three valleys in one cycle of the transient *V*_OC_. The maximal peak of the *V*_OC_ is produced when the fluttering film touches the lower electrode with a large effective contact area (*A*_max_), and the second maximal peak is generated when it taps the upper electrode with an *A*_max_. This difference in the *V*_OC_ is ascribed to downward gravitational force.


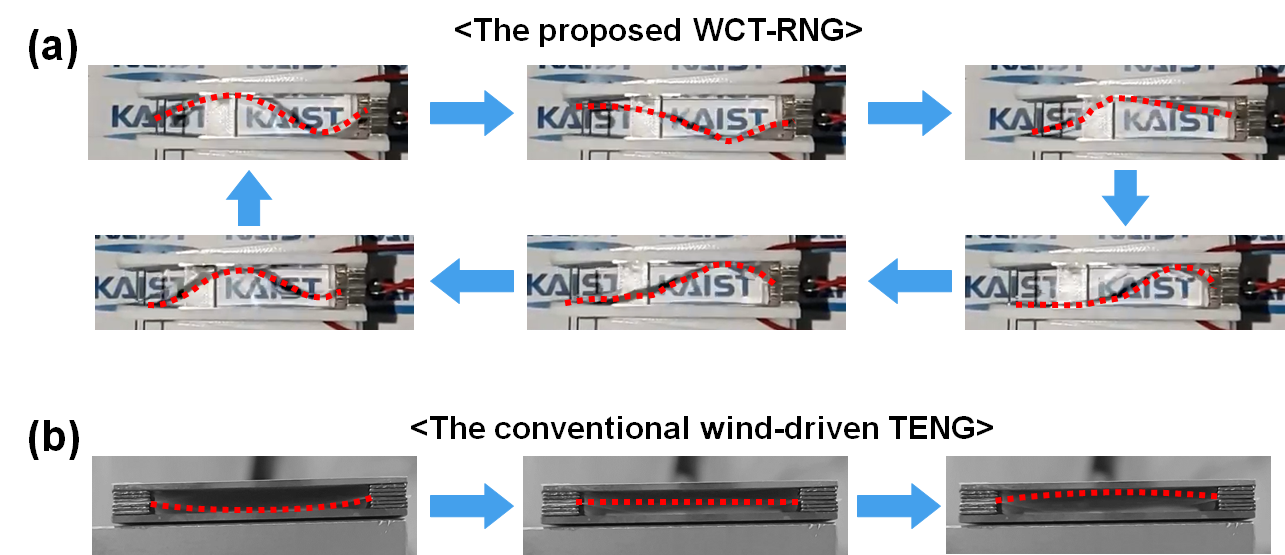


**Figure S3.** Comparison of snapshot photographs between WCT-RNG (experimental group) and 4FW-TENG (control group I). (a) Optical photographs of the WCT-RNG showing a full sine waveform at various positions of the fluttering film in one cycle. (b) Optical photographs of the 4FW-TENG showing a half sine waveform at various positions of the fluttering film in one cycle.

**4. Role of wedge-shaped protrusions and vertical stoppers**

Figure S4 demonstrates the role of the protruded wedges and vertical stoppers (alignment pins). On one hand, when weak wind (gentle breeze) is applied, the left illustration of Fig. S4a shows the fluttering film attached to the lower electrode, while the right illustration of Fig. S4a exhibits the fluttering film separated from the lower electrode. Consequently, the wedge-shaped protrusions assist in lifting the fluttering film easily towards the upper plate and lowering it towards the lower plate. Conversely, when strong wind (windstorm) is applied, the left schematic of Fig. S4b displays the curled-in fluttering film without vertical stoppers, whereas the right illustration of Fig. S4b presents the fluttering film with vertical stoppers, which moves in a sine wave pattern without rolling. As a result, the stoppers prevent the fluttering film from curling inward.

**
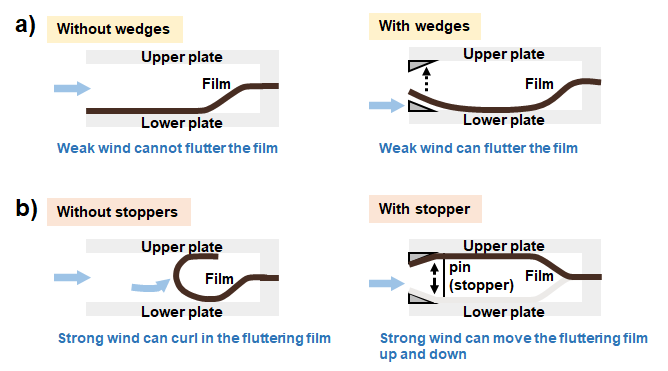
**

**Figure S4.** Role of wedge-shaped protrusions and vertical stoppers. (a) Schematic illustration of stiction between the fluttering film and the lower electrode (left) and non-stiction between them (right) for weak wind. (b) Schematic illustration of curled fluttering film (left) and non-curled fluttering film (right) for strong wind.

**5. Measured open-circuit voltage and short-circuit current**

Figure S5a exhibits the schematic illustration for characterization of *V*_OC_, with its measured values referenced to *V*_GND_. Figure S5b shows the schematic illustration for characterization of *I*_SC_, with its measured values.


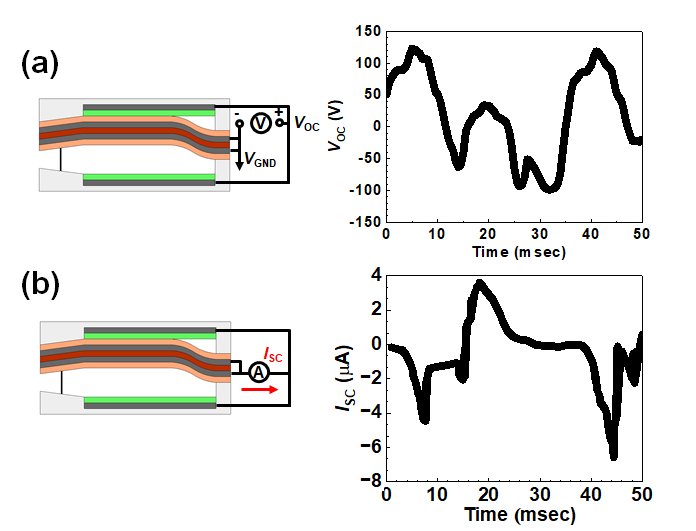


**Figure S5.** (a) Schematic illustration for characterization of open-circuit voltage (*V*_OC_) and its measured values. (b) Schematic illustration for characterization of short-circuit current (*I*_SC_) and its measured values.

**6. Influence of gravitational force on RFW-TENG with decoupling mode**

Figure S5 compares the measured *V*_OC_ for the upright RFW-TENG (control group I) and the upside down RFW-TENG. These experiments were conducted to evaluate whether gravitational force can affect the *V*_OC_ resulting from the vertical movement up and down of the fluttering film. Therefore, the *V*_OC_ was characterized using a decoupled mode, where the upper TENG and the lower TENG have their own independent *R*_load_.

Figure S5a shows a schematic illustration of the upright RFW-TENG, and Figure S5b displays its measured *V*_OC_. The peak value of the *V*_OC_ from the lower TENG is larger than that of the *V*_OC_ from the upper TENG. When the fluttering film moves down, it transfers downward pressure to the lower electrode. In contrast, when the fluttering film moves up, it transfers upward pressure to the upper electrode. Due to the downward gravitational force, the downward pressure of the fluttering film is greater than its upward pressure.

Figure S5c displays a schematic illustration of the flipped RFW-TENG when turned upside down, and Figure S5d presents its measured *V*_OC_. As expected, the peak value of the *V*_OC_ from the upper TENG (initially lower TENG) is larger than that of the *V*_OC_ from the upper TENG (initially lower TENG). This control experiment confirms that the difference in *V*_OC_ between the RFW-TENG in the upright and upside-down positions is indeed attributed to gravitational force.


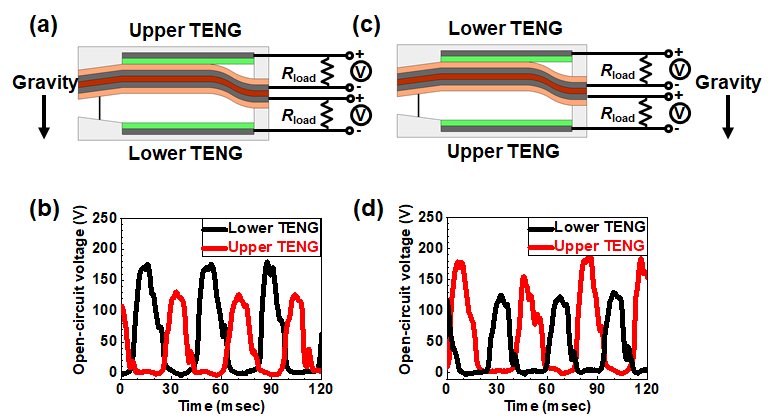


**Figure S6.** Comparison of the RFW-TENG in the upright and upside down positions. (a) Schematic illustration of the upright RFW-TENG. (b) Measured *V*_OC_ for the upright RFW-TENG. (c) Schematic illustration of the RFW-TENG when turned upside down. (d) Measured *V*_OC_ for the upside down RFW-TENG.

**7. Superposition of two sinusoidal signals with two frequencies**

Figure S6 shows the superposition of two ideal sine waves with two frequencies: *f*_1_ of 27.5 Hz and *f*_2_ of 55.0 Hz, and their Fourier transform (FT) data. The superposition of two ideal sine waves describe the superposition of the measured *V*_OC_ and their extracted FT data.


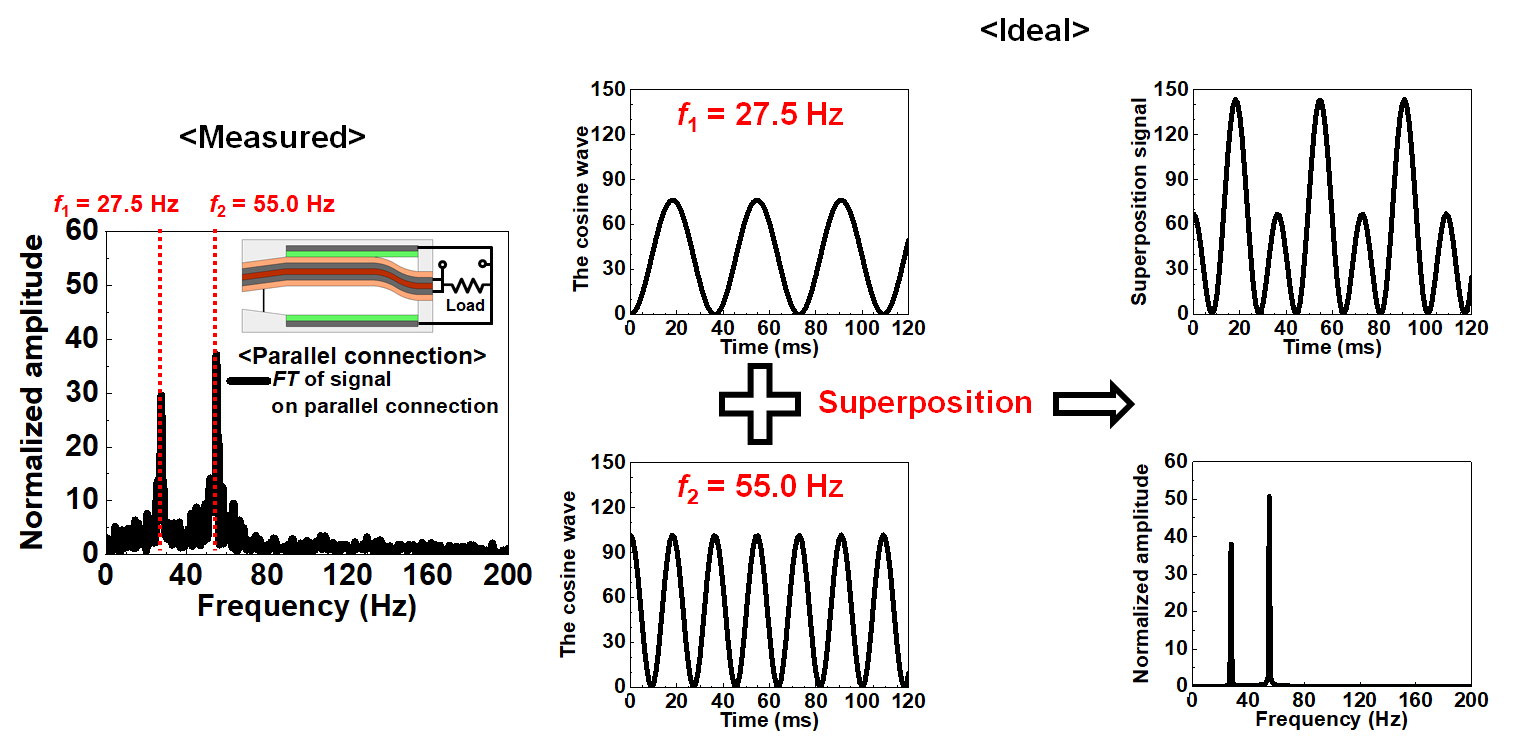


**Figure S7.** Comparison of superposed signals with two frequencies and their FT data between ideal sine waves and measured *V*_OC_.

**REFERENCES**

[1] M, Shoaib, I. Siddiqui, S. Rehman, S. Khan, and L. M. Alhems, Assessment of wind energy potential using wind energy conversion system, *Journal of cleaner production* 216 (2019) 346-360.

[2] S. Rehman, N. Natarajan, M. Vasudevan, and L. M. Alhems, Assessment of wind energy potential across varying topographical features of Tamil Nadu, India, *Energy Exploration & Exploitation* 38 (2020) 175-200.

[3] M. Wadi, and W. Elmasry, Statistical analysis of wind energy potential using different estimation methods for Weibull parameters: a case study, *Electrical Engineering* 103 (2021) 2573-2594.

[4] C. Ozay, and M. S. Celiktas, Statistical analysis of wind speed using two-parameter Weibull distribution in Alaçatı region, *Energy Conversion and Management* 121 (2016) 49-54.

[5] D. K. Kidmo, R. Danwe, S. Y. Doka, and N. Djongyang, Statistical analysis of wind speed distribution based on six Weibull Methods for wind power evaluation in Garoua, Cameroon, *Revue des Energies Renouvelables* 18 (2015) 105-125.
